# Molecules from American Ginseng Suppress Colitis through Nuclear Factor Erythroid-2-Related Factor 2

**DOI:** 10.3390/nu12061850

**Published:** 2020-06-21

**Authors:** Anusha Chaparala, Hossam Tashkandi, Alexander A. Chumanevich, Erin E. Witalison, Anthony Windust, Taixing Cui, Mitzi Nagarkatti, Prakash Nagarkatti, Lorne J. Hofseth

**Affiliations:** 1 Department of OB/GYN, Feinberg School of Medicine, Northwestern University, Chicago, IL 60611, USA; anusha@northwestern.edu; 2 Department of Drug Discovery and Biomedical Sciences, College of Pharmacy, University of South Carolina, Columbia, SC 29208, USA; tashkand@email.sc.edu (H.T.); chumanev@mailbox.sc.edu (A.A.C.); 3 Department of Biology, College of Arts and Sciences, Catawba College, Salisbury, NC 28144, USA; ewhoward12@catawba.edu; 4 Measurement Science and Standards, National Research Council, Ottawa, ON K1A 0R6, Canada; ajwindust@gmail.com; 5 Cell Biology and Anatomy, University of South Carolina School of Medicine, Columbia, SC 29209, USA; Taixing.Cui@uscmed.sc.edu; 6 Department of Pathology, Microbiology, and Immunology, University of South Carolina School of Medicine, Columbia, SC 29209, USA; mitzi.nagarkatti@uscmed.sc.edu (M.N.); PRAKASH@mailbox.sc.edu (P.N.)

**Keywords:** ulcerative, colitis, mice, Nrf2, NFE2L2, panaxynol, falcarinol, inflammatory, inflammation, ginseng

## Abstract

Ulcerative colitis (UC) is a chronic inflammatory bowel disease that affects millions of people worldwide and increases the risk of colorectal cancer (CRC) development. We have previously shown that American ginseng (AG) can treat colitis and prevent colon cancer in mice. We further fractionated AG and identified the most potent fraction, hexane fraction (HAG), and the most potent compound in this fraction, panaxynol (PA). Because (1) oxidative stress plays a significant role in the pathogenesis of colitis and associated CRC and (2) nuclear factor erythroid-2-related factor 2 (Nrf2) is the master regulator of antioxidant responses, we examined the role of Nrf2 as a mechanism by which AG suppresses colitis. Through a series of in vitro and in vivo Nrf2 knockout mouse experiments, we found that AG and its components activate the Nrf2 pathway and decrease the oxidative stress in macrophages (mΦ) and colon epithelial cells in vitro. Consistent with these in vitro results, the Nrf2 pathway is activated by AG and its components in vivo, and Nrf2^-/-^ mice are resistant to the suppressive effects of AG, HAG and PA on colitis. Results from this study establish Nrf2 as a mediator of AG and its components in the treatment of colitis.

## 1. Introduction

Inflammatory bowel disease (IBD), which includes Ulcerative colitis (UC) and Crohn’s disease (CD), debilitates approximately 2.6 million people in North America, and this number is on the rise [1]. In particular, the relative risk for colorectal cancer (CRC) in patients with UC is tenfold greater than the general population [2]. The etiology of UC remains to be fully elucidated but involves interactions among genetic, environmental, and immune factors, leading to uncontrolled abnormal immune responses in the intestinal mucosa [3]. Chronic intestinal inflammation is associated with enhanced reactive oxygen species (ROS) production, and the consequent oxidative stress plays a critical role in the pathophysiology of IBD in both animals and humans [4,5,6]. Prevention of IBD has been shown by transgenic overexpression of endogenous antioxidant genes and administration of antioxidant compounds [7,8,9,10]. Treatment strategies for UC (and the prevention of CRC) reduce periods of active disease and maintain remission, but patients become refractory, and there are dangerous side effects like cancer, infection, sepsis, hepatitis, and death [11,12,13,14]. For that reason, 40%–50% of IBD patients use some form of complementary and alternative medicine (CAM) [15,16]. Identifying new treatments that have minimal toxicity to treat the disease and prevent colon cancer, therefore, remains a high priority.

Through many years of experimentation, we have demonstrated that American ginseng (AG) suppresses colitis in mice without toxicity [17,18,19]. In further delineating the active components of AG, a fraction of AG was derived from extraction with n-hexane as the solvent (and therefore designated as the hexane fraction of American ginseng [HAG]), which was particularly potent in suppressing colitis and preventing CRC in mice [20,21,22]. We have also observed that panaxynol (PA), an abundant molecule in this fraction, suppresses colitis in mice. The next logical step was to explore the molecular mechanisms of the beneficial effects of AG and its components on the treatment of UC.

Nuclear factor erythroid-2-related factor 2 (Nrf2), a master regulator of the antioxidant response element, is a member of the cap’n collar family of basic region-leucine zipper transcription factor [23]. As a transcription factor, Nrf2 promotes the expression of phase II antioxidant enzymes that include, but is not limited to, nicotinamide adenine dinucleotide (phosphate) hydrogen (NAD[P]H) quinone oxidoreductase-1 (NQO1) and heme oxygenase-1 (HO-1), which protect against ROS. The precise mechanisms of Nrf2 activation are not fully understood, but it is widely accepted that Keap1 is a key regulator of Nrf2. Knowing that (1) Nrf2 is a key initiator of antioxidant response required for the treatment of UC [7,8,9,10]; (2) AG suppresses colitis [17,18,19,20,21,22]; and (3) ginseng and its components (including PA) can induce Nrf2 [24,25,26,27], we hypothesized that Nrf2 is at the crossroads of the protective action of AG, HAG, and PA against colitis. Here we show results that are consistent with this hypothesis.

## 2. Materials and Methods

### 2.1. Cell Culture

All cell lines were maintained in cell culture media supplemented with 10% fetal bovine serum (FBS), 1% penicillin, and were kept at 37 °C in a humidified incubator with 5% CO_2_;. ANA-1 cells were grown in Dulbecco’s modified Eagle’s medium (DMEM, Hyclone, Logan, UT), and HCT-116 cells were cultured in Roswell Park Memorial Institute (RPMI)-1640 media. For the activation of ANA-1 cells, the cells were plated in 100 mm dishes, allowed to attach overnight, and were treated with 10 ng/mL interferon (IFN)-γ in 2 mL media (R&D Systems, Minneapolis, MN, USA) for 12 h. For co-culture experiments, 1 × 10^6^ HCT-116 cells were plated and allowed to attach overnight in 6-well dishes. Then, they were pretreated with AG (100 µg/mL), HAG (100 µg/mL), or PA (0.25 µg/mL) for 8 h. Activated ANA-1 cells were then plated at 3 × 10^6^ cells/well in transwell inserts (cat. # 2018-11, Greiner bio-one; Kremsmünster, Austria), which were then placed in the 6-well dishes to facilitate the exchange of cytokines and other cellular secretions. The 6-well dishes have a growth area of 9.5 cm^2^, and the transwell inserts have a growth area of 4.524 cm^2^.

### 2.2. ROS Detection

ANA-1 mΦ were pre-treated with AG (100 µg/mL), HAG (100 µg/mL), or PA (0.25 µg/mL) for 12 h in 6-well dishes. The media was replaced with media containing IFN-γ (10 ng/mL), the cells were treated for 1, 2, or 3 h, and were then processed according to the instructions of the Superoxide/Total Oxidant Species Chemiluminescent kit (lot # 101214A; Applied Bioanalytical labs). Briefly, the cells were washed with phosphate buffered saline (PBS) and resuspended in Hank’s balanced salt solution (HBSS). Luminol and enhancer solutions were added before measuring the luminescence in a luminometer at 425 nm. Luminometer quantifies an oxidative burst through the reaction of luminol with ROS to produce a luminophore. Luminescence intensity is proportional to the number of reactive species in the sample and is quantified as relative light units. 

### 2.3. Western Blot

For the gel electrophoresis, proteins were mixed with Novex Tris-Glycine SDS Sample buffer (LC2676; Thermo Fisher Scientific, Waltham, MA, USA) for a final concentration of 2 µg/µL. Enough mixture of protein and sample buffer was obtained to ensure 30 µg of protein were loaded in Novex WedgeWell 4%–20% Tris-Glycine Mini Gels, 15-well (XP04205BOX; Thermo Fisher Scientific, Waltham, MA, USA). The primary antibodies, HO-1 (ab13248; Abcam, Cambridge, UK) and GAPDH (5174S; Cell Signaling, Danvers, MA, USA), were diluted to 1:1000 and incubated with the membrane overnight at 4 °C. Horseradish peroxidase-conjugated anti-rabbit secondary antibodies (7074S; Cell Signaling, Danvers, MA, USA) at 1:2000 dilution were incubated at room temperature for 1 h. The Western blot signal was detected by Pierce ECL Western Blotting Substrate (Thermo Fisher Scientific, Waltham, MA, USA) and imaged using Bio-Rad ChemiDoc Imager (17001402; Bio-Rad, Hercules, CA, USA).

### 2.4. Immunofluorescence

Cells (5 × 10^5^ per well) were seeded into six well plates containing a coverslip. The next day, cells were rinsed with ice-cold PBS and fixed with 4% paraformaldehyde for 10 min at room temperature, followed by permeabilization with 0.1% sodium citrate with 0.1% Triton X-100. The cells were incubated with Nrf2 antibody at 1:1000 dilution (12721S; Cell Signaling, Danvers, MA, USA) for 1 h at room temperature. The cells were then washed with cold PBS three times (5 min each) and incubated with Alexa 514-labeled anti-rabbit secondary antibody (1:1000) (A11061; Thermo Fisher Scientific, Waltham, MA, USA) at room temperature for 1 h. The cells were then incubated with conjugated actin antibodies for 15 min. After washing with PBS, the coverslips were mounted onto the slides with ProLong Diamond Antifade Mountant with DAPI (P36962; Thermo Fisher Scientific, Waltham, MA, USA). Standard immunofluorescence methods were used as described previously [28]. Imaging was performed using EvosFL fluorescence microscope (Life Technologies, Carlsbad, CA, USA) at 40× objective magnification. Fluorescence intensities from images of six randomly selected microscopic fields of cells were semi-quantitatively analyzed by densitometry (ImageJ software, NIH).

### 2.5. Immunohistochemistry

For immunohistochemical staining, formalin-fixed, paraffin-embedded serial sections of mouse colon tissues were incubated overnight at 4 °C with the following primary antibodies: anti-HO-1 (ab13248; Rabbit Polyclonal, diluted 1:2000; Abcam, Cambridge, UK), 4-Hydroxynonenal (4-HNE) (ab46545; Rabbit Polyclonal, diluted 1:1000; Abcam, Cambridge, MA, USA), or cyclooxygenase-2 (COX2) (Rabbit Polyclonal, diluted 1:1000; 60126; Cayman Chemical Company, Ann Arbor, MI, USA). To ensure even staining and reproducibility, sections were incubated by slow rocking using the Antibody Amplifier (AA1; ProHisto, LLC, Columbia, SC, USA). Sections were processed with EnVision+ System-HRP kit according to the kit protocols (K4011; Dako Cytomation, Carpinteria, CA, USA). The chromogen was diaminobenzidine, and sections were counterstained with 2% methyl green. The negative control was carried out in the absence of a primary antibody. Intensity and degree of specific staining were evaluated independently by two blinded investigators. Immunohistochemistry score was quantified as described previously [22].

### 2.6. RNA Isolation and RT-qPCR

Cells were lysed using Trizol, and RNA was isolated using the RNeasy mini kit (#74104; Qiagen, Hilden, Germany) according to the instructions. RNA concentrations were measured using NanoDrop 2000 spectrophotometer (Thermo Fisher Scientific, Waltham, MA, USA). One microgram of RNA was used for cDNA synthesis using iScript cDNA synthesis kit (#1708890; Bio-rad, Hercules, CA, USA). The final product was diluted 1:10 for qPCR. qPCR was performed using iTaq Universal SYBR Green Supermix (1725121; Bio-rad, Hercules, CA, USA). Fifty nanograms of cDNA were used with 500 nM of each primer. qPCR was carried out on CFX384 Touch Real-Time PCR Detection System (1855485; Bio-rad, Hercules, CA, USA). DNA polymerase activation and denaturation were done for 30 s at 95 °C, 40 cycles of denaturation for 5 s each at 95 °C, and annealing was done for 30 s at 60 °C. Primer design and selection was done using Primer-BLAST tool provided by the NIH. The list of primers used is as follows in the order of forward then reverse in 5′-3′ orientation:

#### 2.6.1. Mouse Primers

HO-1: AAGCCGAGAATGCTGAGTTCA, GCCGTGTAGATATGGTACAAGGA

Nrf2: TCTTGGAGTAAGTCGAGAAGTGT, GTTGAAACTGAGCGAAAAAGGC

GAPDH: AGGTCGGTGTGAACGGATTTG, TGTAGACCATGTAGTTGAGGTCA

#### 2.6.2. Human Primers

HO-1: CCACCTGTTAATGACCTTGCC, CACCGGACAAAGTTCATGGC

Nrf2: TCAGCGACGGAAAGAGTATGA, CCACTGGTTTCTGACTGGATGT

GAPDH: AATCCCATCACCATCTTCCA, TGGACTCCACGACGTACTCA

### 2.7. Dextran Sulfate Sodium (DSS) Mouse Model

All animal experimental protocols were performed in accordance with the Institutional Animal Care and Use Committee (IACUC) at the University of South Carolina under an approved protocol (2375-101235-081417). We followed our previously described protocol for the DSS (MP Biomedicals, Solon, OH: 36,000–50,000 mw) mouse model of colitis [21,22]. Briefly, 8- to 10-week-old C57BL/6 wild-type (WT) and C57BL/6 Nrf2^−/−^ (Nrf2^−/−^) (The Jackson Laboratory, B6.129X1-Nfe2l2tm1Ywk/J) mice received either water ad libitum or 2% DSS for two weeks. All mice were on an AIN93 M diet. During days 7–14, vehicle (PBS), AG (75 mg/kg/day), HAG (75 mg/kg/day), or PA (1 mg/kg/day) was dissolved in 100 μL of PBS and administered daily by oral gavage (per os, PO). The concentrations for AG and HAG were chosen to contain the same amount of PA given in this experiment. On the day of the sacrifice, stool consistency (0—fully formed stool; 2—loose stool; 4—diarrhea) and blood in the stool (0—no blood; 2—detected using Hemoccult; 4—rectal bleeding) were scored, and these measurements were used along with the weight difference in mice from the beginning to the end of the experiment (0 = no weight loss; 1 = 0%–5% weight loss; 2 = 6%–10% weight loss; 3 = 11%–15% weight loss; 4 = 16%–20% weight loss) to calculate the clinical disease index (CDI). Blood in the stool was detected using Beckman Coulter Hemoccult Fecal Occult Blood Slide Test System (SK-60151; Fisher Scientific, Waltham, MA, USA) fecal immunochemical test. Mice were sacrificed on day 14, 24 h after the last treatment; colons were fixed overnight in formalin after they were cut longitudinally, and their lengths were measured. The colons were then embedded in paraffin for sectioning.

### 2.8. Quantifying Inflammation

Paraffin-embedded colons were serially sectioned (5 µm), and one section from each mouse was stained with hematoxylin and eosin (H&E). The H&E-stained slides were blindly examined under a microscope by two independent investigators for histopathological changes and scored according to the system previously described [22,28,29]. The histology score for inflammation accounts for four parameters: (1) inflammation severity (0 = no inflammation, 1 = minimal, 2 = moderate, and 3 = severe); (2) inflammation extent (0 = no inflammation, 1 = mucosa only, 2 = mucosa and submucosa, and 3 = transmural); (3) crypt damage (0 = no crypt damage, 1 = one-third of crypt damaged, 2 = two-thirds damaged, 3 = crypts lost and surface epithelium intact, and 4 = crypts lost and surface epithelium lost); and (4) percentage area of involvement (0 = 0% involvement, 1 = 1%–25%, 2 = 26%–50%, 3 = 51%–75%, and 4 = 76%–100%). The scores for the first three parameters were added, and the sum was multiplied by the fourth parameter, giving a range of scores between 0–40.

### 2.9. Statistical Analysis

Data are expressed as a mean ± standard error of the mean. Mean differences between the groups were compared by one-way analysis of variance (ANOVA), followed by Dunnett’s multiple comparison tests. A *p*-value of ≤0.05 was chosen for significance.

## 3. Results

### 3.1. American Ginseng and Its Derivatives Suppress Oxidative Stress and Activate Nrf2 Pathway in Macrophages

**Figure 1:**
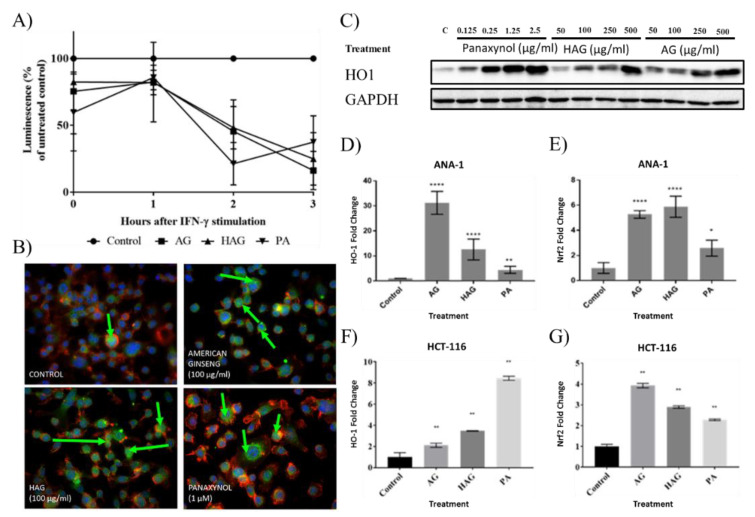
American ginseng (AG), hexane fraction of American ginseng (HAG), and panaxynol (PA) activate Nrf2 pathway and decrease reactive oxygen species (ROS) in vitro. (**A**) An oxidative burst in ANA-1 mouse mΦ is suppressed by pretreatment with AG (100 μg/mL), HAG (100 μg/mL), and PA (0.25 μg/mL). The protocol is described in the Methods section. (**B**) AG, HAG, and PA induce translocation of Nrf2 into the nucleus. ANA-1 cells were treated with AG (100 μg/mL), HAG (100 μg/mL), and PA (0.25 µg/mL = 1 µM) for 12 h. Representative images of immunofluorescence (n = 3). Green arrows indicate nuclei with Nrf2 expression. (**C**) AG, HAG, and PA increase the expression of HO-1. Western blot image of ANA-1 cells treated with indicated doses of AG, HAG, and PA for 12 h. C—control sample: non-treated ANA-1 cells. (**D**,**E**) Twelve-hour incubation with AG (100 μg/mL), HAG (100 μg/mL), and PA (0.25 µg/mL) increases the expression of HO-1 (**D**) and Nrf2 (**E**) in ANA-1 cells. RT-qPCR data is cumulative of three separate experiments. (**F**,**G**) AG, HAG, and PA activate Nrf2 pathway in HCT-116 cells in the presence of activated mΦ. HCT-116 cells were pretreated with AG (100 μg/mL), HAG (100 μg/mL) and PA (0.25 µg/mL) and co-cultured with activated ANA-1 (10 ng/mL IFN-γ) for 3 h and separated for qPCR. *p*-values—*—>0.05, **—>0.005, ***—>0.001, ****—>0.0001. Values represent the mean ±S.D. The significance is compared with the control group.

We have previously shown that AG suppresses ROS in vitro [18,19]. Consistent with and advancing those findings, we show here that ANA-1 cells pre-treated with AG, HAG, and PA produced significantly decreased amounts of the total ROS associated with IFN-γ activation (Figure 1A). Given that Nrf2 is important in response to inflammation, we looked at the translocation of Nrf2 into the nucleus, as well as activation of a key target gene activated by this transcription factor using AG, HAG, or PA. Immunofluorescence data shows that Nrf2 was localized to the nucleus upon treatment with AG, HAG, or PA, indicating its activation (Figure 1B). HO-1, a target of Nrf2, was also upregulated by AG, HAG, and PA treatment of ANA-1 mΦ, as shown by Western blot and RT-qPCR (Figure 1C,D). Furthermore, AG, HAG, and PA treatments elevated the expression of the Nrf2 gene (Figure 1E). To check the activation of Nrf2 in epithelial cells in an inflammatory environment, we co-cultured HCT-116 cells with activated ANA-1 cells for 3 h after pretreating the HCT-116 cells for 8 h with AG, HAG, or PA. RT-qPCR data indicates that both Nrf2 and HO-1 were overexpressed when HCT-116 cells were pre-treated with AG, HAG, or PA, even in the presence of proinflammatory ANA-1 mΦ (Figure 1F,G).

### 3.2. American Ginseng and Its Derivatives Suppress Oxidative Stress and Activate Nrf2 Pathway In Vivo

**Figure 2:**
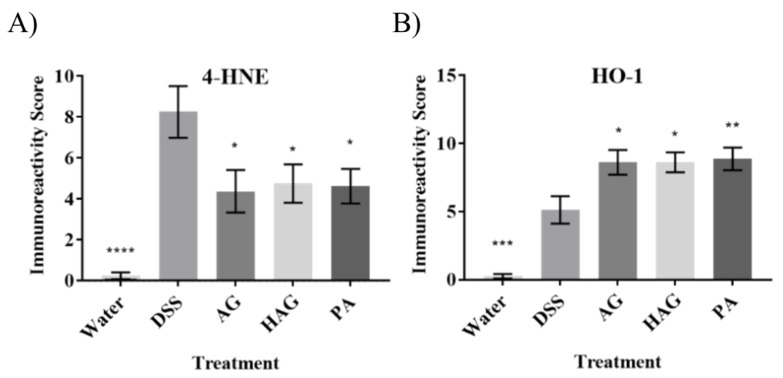
AG, HAG, and PA activate Nrf2 pathway and decrease ROS in vivo. Effect of AG, HAG, and PA on oxidative stress and Nrf2 pathway activation. Dextran Sodium Sulphate (DSS)-induced colitis mice were treated with AG (75 mg/kg/day), HAG (75 mg/kg/day), or PA (1 mg/kg/day). Colons from these mice were probed for (**A**) 4-HNE and (**B**) HO-1 to indicate oxidative stress and Nrf2 pathway activation, respectively. Values represent the mean ±S.E. N = 8. The significance is compared with the DSS-only group. *p*-values—*—>0.05, **—>0.005, ***—>0.001, ****—>0.0001.

To evaluate if Nrf2 is activated by the AG, HAG, and/or PA in vivo, the colons of mice treated with DSS or DSS combined with AG, HAG, or PA were probed for 4-HNE and HO-1. Since 4-HNE is known to activate Nrf2 [29] and is also a marker of inflammation, we probed for this endpoint. Figure 2A shows induced expression of 4-HNE in the DSS-treated group and its suppression when mice also consumed AG, HAG or PA. We have previously shown that AG and HAG suppress colitis in mice [18,19,21,22,30]. Consistent with our previous findings, AG, HAG, and now PA suppress colitis in DSS-treated mice (Figure 2A). Figure 2B shows induction of HO-1 in colitis (consistent with previous findings [31,32]) and further induction with administration of AG, HAG, or PA.

### 3.3. American Ginseng and Its Derivatives Suppress Colitis by Activating Nrf2 Pathway

**Figure 3:**
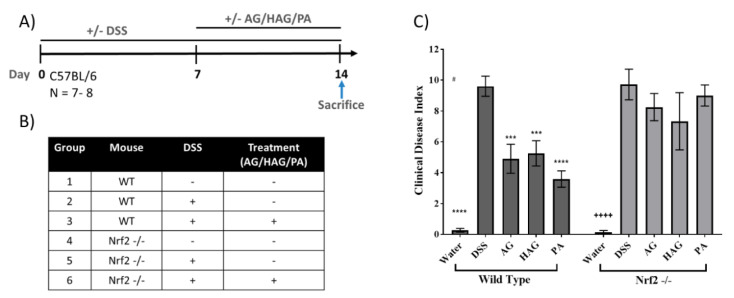
AG, HAG, and PA decrease the clinical disease index (CDI) in WT mice but not Nrf2^-/-^ mice. (**A**,**B**) Schematic and groups of the experiment. (**C**) Effect of AG (75 mg/kg/day), HAG (75 mg/kg/day), and PA (1 mg/kg/day) on the clinical disease index, which accounts for weight loss, blood in stool, and stool consistency. Values represent the mean ±S.E. Significance is compared with the DSS only sub-group within WT and Nrf2^-/-^ groups. *p*-values—*>0.05, **—>0.005, ***—>0.001, ****—>0.0001.

**Figure 4:**
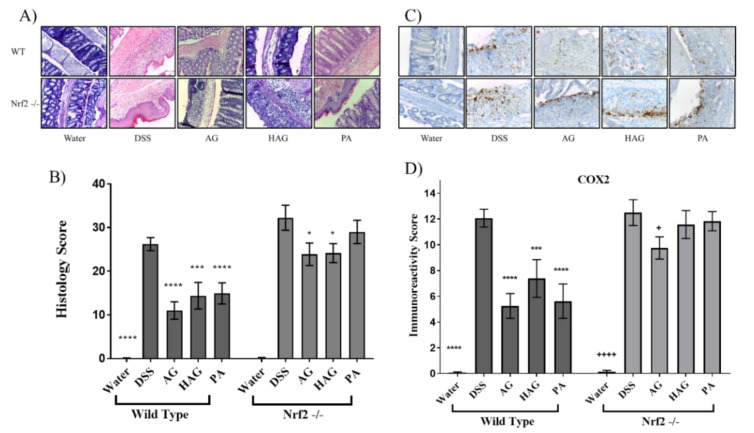
AG, HAG, and PA decrease the inflammation in WT mice but not Nrf2^-/-^ mice. Seven to ten mice from each group from Figure 3 were euthanized on day 14, and colons were harvested. (**A**,**B**) Effects of AG, HAG, and PA on the colon histology score in the acute DSS colitis model. The histology score was determined as described in Materials and Methods. Values represent the mean ±S.E. of the mean. Representative H&E-stained colons are shown for each group (**A**). Sections of the colon were probed for cyclooxygenase-2 (COX2). (**C**) Representative IHC images probed for COX2. (**D**) Immunoreactivity score. * Significant difference from the DSS group *p*-values *—>0.05, **—>0.005, ***—>0.001, ****—>0.0001. A and C are representative images (400× magnification).

With evidence indicating Nrf2 as a mediator of suppression of colitis by AG, HAG, and PA, we tested the hypothesis that these ingredients would suppress colitis in WT but not in Nrf2^−/−^ mice exposed to DSS (Figure 3). As expected, AG, HAG, and PA suppressed DSS-induced colitis in WT but not Nrf2^−/−^ mice (Figure 3C and Figure 4A,B). We also tested immunoreactivity of COX2, a proinflammatory enzyme that has elevated protein expression during inflammation. Again, AG, HAG, and PA each effectively decrease COX2 expression in WT mice but not the Nrf2^−/−^ mice (Figure 4C,D).

## 4. Discussion

IBD, by definition, is described as a complex chronic colon inflammation condition. Therefore, there is no surprise that antioxidant-based approaches have shown efficacy in treating IBD [33]. Current FDA-approved therapies involve treatment with 5-aminosalicylic acid and its analogs, as well as glucocorticosteroids and anti-TNFα biologicals. Such treatment reduces periods of active disease and helps to maintain remission. Although these drugs have modest beneficial effects, patients become refractory, and there are significant side effects [34]. This underscores the importance of developing more effective IBD therapies. To this end, we have explored the potency of AG, a natural compound, in the treatment of colitis and identified the bioactive components that could be responsible for AG’s efficacy. The identification of a single, active component would bring AG a step closer to being used as a mainstream medicine. In this context, it makes sense and would be significant to explore the molecular mechanisms of AG-mediated beneficial effects on the treatment of IBD.

In this study, we evaluated the antioxidant response and activation of the Nrf2 signaling pathway in macrophage cell lines (in vitro) and mouse colon (in vivo) upon treatment with AG or its components. Treatment with either AG, HAG, or PA has activated HO-1 in both ANA-1 macrophage cell lines and mouse colons in the DSS-induced colitis model. They also induced the translocation of Nrf2 into the nucleus, indicating its activation. Furthermore, we observed that each of these treatment compounds also caused suppression of both ROS production in vitro and the expression of 4-HNE in vivo. Taken together, such an inhibition indicates decreased oxidative stress. Upon confirming that AG and its components activate the Nrf2 pathway, we used Nrf2^−/−^ mice to investigate if the Nrf2 pathway is essential for the treatment of colitis. As predicted, AG and its components were not very effective in the Nrf2^−/−^ mice as evidenced by the inflammation scores and expression of COX2.

**Figure 5:**
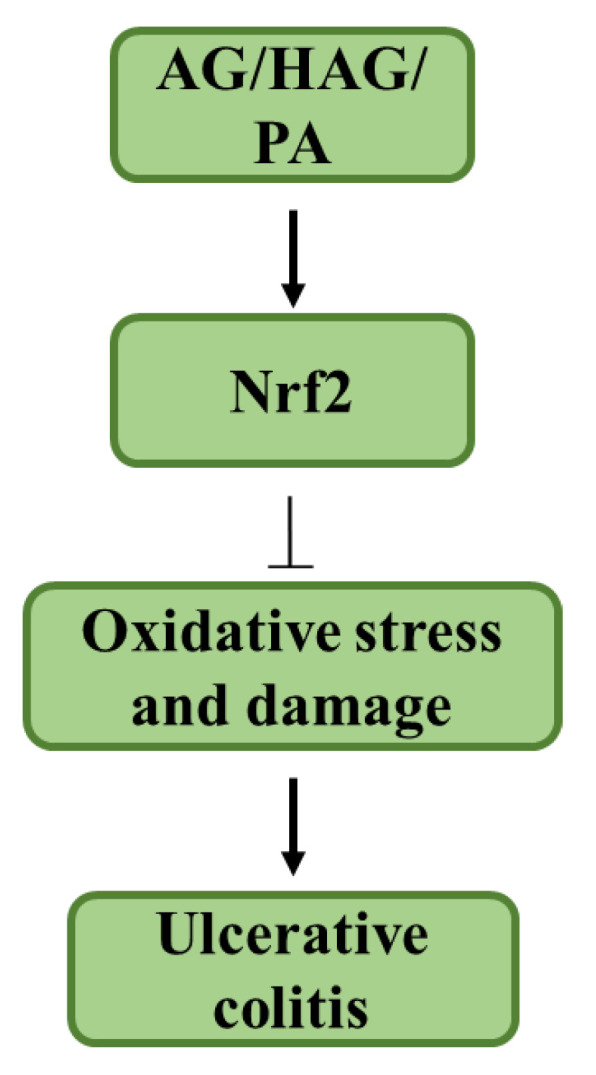
AG, HAG, and PA suppress colitis by activation of the Nrf2 pathway. Schematic representing the conclusions: oxidative stress is one of the factors that play a significant role in the progression of colitis. AG, HAG, and PA activate the transcription factor, Nrf2, which in turn activates antioxidant genes that decrease oxidative stress, thereby suppressing colitis.

Though the effects of AG and its components have been diminished considerably, there is still a decrease in inflammation with AG treatment of Nrf2^−/−^ mice. This could be due to the effect of p53, which also plays an important role in the activation of antioxidant mechanisms like the synthesis of GSH and NADPH generation [35,36]. We have previously shown that the mechanism of action of AG in the treatment of colitis is p53 dependent, but the mechanism of HAG is only partially dependent on p53 [19,21]. Apart from possessing antioxidant properties, Nrf2 has also been shown to negatively regulate the expression of pro-inflammatory genes that are induced in M1 pro-inflammatory mΦ [37]. This is consistent with the decreased expression of ROS, the proinflammatory markers, and could be an additional mechanism by which Nrf2 activation helps in the treatment of colitis that we would explore in the future (Figure 5).

Because Nrf2 is involved in colitis and associated with colon cancer, we believe it would be significant to examine the ability of AG, HAG, and PA to target Nrf2 as a mechanism for the protection of colitis and colon cancer [38,39,40]. Such studies will not only better elucidate the mechanisms by which AG, HAG, and PA work in this disease, but also (1) identify other diseases where Nrf2 is dysregulated and AG, HAG, and individual constituents may be beneficial, such as obesity, neurodegenerative diseases, and cardiovascular disease [41,42,43]; (2) identify other CAMs or small molecules that target Nrf2 that may be beneficial to patients with colitis at a high colon cancer risk, such as triterpenoids that target Nrf2 and also suppress experimental colitis [44,45]; and (3) identify epigenetic biomarkers of Nrf2 regulation and attenuation of inflammation.
